# Chloroplast genome and phylogenetic analyses of *Morus alba* (Moraceae)

**DOI:** 10.1080/23802359.2019.1673242

**Published:** 2020-06-01

**Authors:** Shui-Lian He, Yang Tian, Yang Yang, Chong-Ying Shi

**Affiliations:** a College of Horticulture and Landscape, Yunnan Agricultural University, Kunming, China; b Yunnan Key Laboratory of Biomass Big Data, Yunnan Agricultural University, Kunming, China; c College of Science, Yunnan Agricultural University, Kunming, China; d Institute of Food Science and Technology, Yunnan Agricultural University, Yunnan, China

**Keywords:** *Morus alba*, medicinal and edible plant, chloroplast genome, phylogenetic analysis

## Abstract

*Morus alba* is an important medicinal plant that is used to treat human diseases. The complete chloroplast (cp) genome of *M. alba* was assembled based on the Illumina sequencing reads. The cp genome of *M. alba* was 159,290 bp and contained two short inverted repeat regions (25,690 bp) which were separated by a small single copy region (19,845 bp) and a large single copy region (88,065 bp). The cp genome encodes 111 unique genes, including 77 protein-coding genes, 30 transfer RNA genes and four ribosomal RNA genes. The topology of the phylogenetic tree showed that *M. alba* is closely clustered with species *M. cathayana* and *M. mongolica*.

*Morus alba* L., named mulberry tree, is a deciduous woody shrub in the family Moraceae and widely cultivated in China, Korea, India, and Japan (Umate 2010). Its fruit is edible for human and its leaf is food of Silkworm. *Morus alba* L. is also an important medicinal plant that is used to treat human diseases. The leaf, branch, and root of *Morus* can be applied as antidiabetic, antioxidant, and anti-inflammatory medicines, respectively (Zhu et al. 2019). In order to clarify the taxonomical position of *M. alba* in Moraceae, We applied the Illumina technology to sequence, assemble and annotate the whole chloroplast genome of *M. alba*. The resultant data have been made publicly available as a resource for genetic information for *Morus* species, and will provide a valuable plastid genomic resource for the future genetic and phylogenetic studies about *M. alba.*

The fresh leaves of *M. alba* were collected from the field of Kunming (25.20°N, 102.86°E). The voucher specimen was deposited at Herbarium of Yunnan Agricultural University (No. 2019HSL004). Total genomic DNA was isolated from fresh leaves using a DNeasy Plant Mini Kit (QIAGEN, Valencia, California, USA) according to the manufacturer’s instructions to construction chloroplast DNA libraries. The Illumina sequencing was conducted by Biomarker Technologies Inc. (Beijing, China). Resultant clean reads were assembled using GetOrganelle pipeline (https://github.com/Kinggerm/GetOrganelle). The genome was automatically annotated by using the CpGAVAS pipeline (Liu et al. 2012) and start/stop codons and intron/exon boundaries were adjusted in Geneious R11.0.2 (Biomatters Ltd., Auckland, New Zealand). All the contigs were checked against the reference genome of *Morus mongolica* (NC025772)

The complete chloroplast genome of *M. alba* was 159,290 bp in length (Genbank accession number: MN102359). It was the typical quadripartite structure and contained two short inverted repeat (IRa and IRb) regions (25,690 bp bp) which were separated by a small single copy (SSC) region (19,845 bp) and a large single copy (LSC) region (88,065 bp). The cp genome encodes 111 unique genes, including 77 protein-coding genes, 30 transfer RNA (tRNA) genes and four ribosomal RNA (rRNA) genes. Eighteen gene species are partially or completely duplicated, including seven PCG (*ndhB*; *ndhF*; *rpl2*; *rpsl23*; *rps7*; *ycf1*; *ycf2*), seven tRNA (*trnI-GAU*, *trnA-UGC*, *trnL-CAA*, *trnI-CAU*, *trnR-ACG*, *trnV-GAC*, *trnN-GUU*) and all four rRNA (4.5S, 5S, 16S & 23S rRNA). The overall GC content of the cp genome was 36.2%, while that of LSC, SSC and IR regions was 33.8%, 29.2% and 42.9%, respectively.

A total of 11 cp genome sequences were selected to infer the phylogenetic relationships among the main representative species of Moraceae with *Magnolia alba* (*NC037005,* Magnoliaceae) as outgroup. The combined datasets based on plastid genomes were aligned by MAFFT v7.307 (Katoh and Standley 2013). A neighbour-joining (NJ) phylogenetic tree was constructed in Geneious 11.1.5 (Kearse et al. 2012) with the Tamura-Nei genetic distance model, and a total of 1000 bootstrap replicates were performed. The topology of the phylogenetic tree showed that *M. alba* has a close relationship with species *M. cathayana* and *M. mongolica*. (Figure 1). The complete cp genome information reported in this study will be a valuable resource for future studies on genetic diversity, taxonomy and phylogeny of the Moraceae.

**Figure 1.**
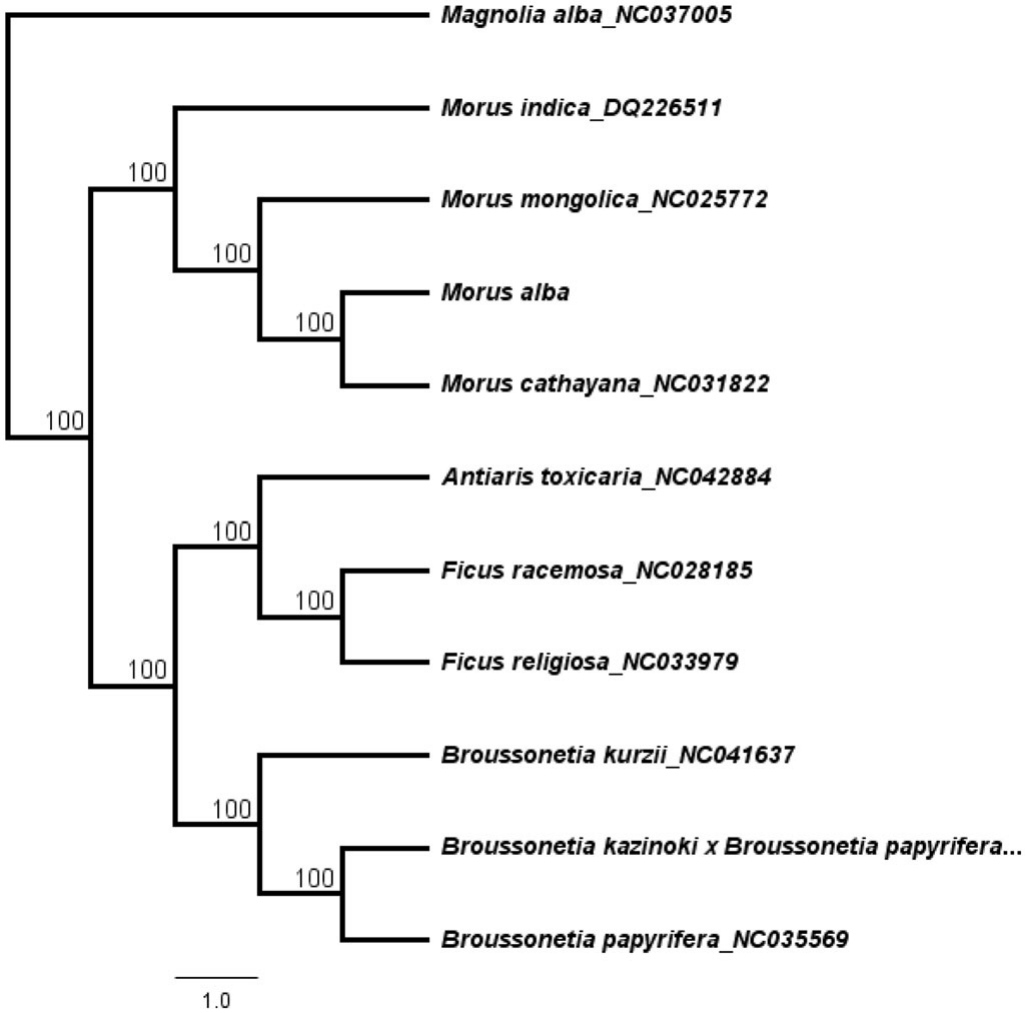
The neighbour-joining (NJ) phylogenetic tree based on 11 complete chloroplast genome sequence. Numbers at the right of nodes are bootstrap support values.
